# ‘Confidence and fulfillment’: a qualitative descriptive study exploring the impact of palliative care training for long-term care physicians and nurses

**DOI:** 10.1177/26323524241235180

**Published:** 2024-03-05

**Authors:** Ashlinder Gill, Lynn Meadows, Jessica Ashbourne, Sharon Kaasalainen, Sandy Shamon, José Pereira

**Affiliations:** Division of Palliative Care, Department of Family Medicine, McMaster University, 5th Floor, 100 Main Street West, Hamilton, ON, Canada L8P 1H6; Department of Community Health Sciences, Cumming School of Medicine, University of Calgary, Calgary, AB, Canada; Division of Palliative Care, Department of Family Medicine, McMaster University, Hamilton, ON, Canada; Faculty of Health Sciences, Division of Palliative Care, Department of Family Medicine, School of Nursing, McMaster University, Hamilton, ON, Canada; Division of Palliative Care, Department of Family Medicine, McMaster University, Hamilton, ON, Canada; Temmy Latner Centre for Palliative Care, Toronto, ON, Canada; Division of Palliative Care, Department of Family Medicine, McMaster University, Hamilton, ON, Canada; Pallium Canada, Ottawa, ON, Canada

**Keywords:** education, interprofessional education, long-term care, palliative care

## Abstract

**Objective:**

To explore the impact of a 2-day, in-person interprofessional palliative care course for staff working in long-term care (LTC) homes.

**Methods:**

A qualitative descriptive study design was employed. LTC staff who had participated in Pallium Canada’s Learning Essential Approaches to Palliative Care LTC Course in Ontario, Canada between 2017 and 2019 were approached. Semi-structured interviews were conducted, using an online videoconferencing platform in mid-2021 in Ontario, Canada. These were done online, recorded, and transcribed. Data were coded inductively.

**Results:**

Ten persons were interviewed: four registered practical nurses, three registered nurses, one nurse practitioner, and two physicians. Some held leadership roles. Participants described ongoing impact on themselves and their ability to provide end-of-life (EOL) care (micro-level), their services and institutions (meso-level), and their healthcare systems (macro-level). At a micro-level, participants described increased knowledge and confidence to support residents and families, and increased work fulfillment. At the meso-level, their teams gained increased collective knowledge and greater interprofessional collaboration to provide palliative care. At the macro level, some participants connected with other LTC homes and external stakeholders to improve palliative care across the sector. Training provided much-needed preparedness to respond to the impact of the COVID-19 pandemic, including undertaking advance care planning and EOL conversations. The pandemic caused staff burnout and shortages, creating challenges to applying course learnings.

**Significance of results:**

The impact of palliative care training had ripple effects several years after completing the training, and equipped staff with key skills to provide care during the COVID-19 pandemic. Palliative care education of staff remains a critical element of an overall strategy to improve the integration of palliative care in LTC.

## Introduction

Most residents living in long-term care (LTC) homes have life-limiting conditions that compromise their quality of life.^1^ Moreover, up to 20% of LTC residents in Canada die annually.^1,2^ With the goal of optimizing their quality of life and reducing end-of-life (EOL) suffering, many residents would therefore benefit from palliative care, preferably initiated soon after admission. The term ‘palliative care approach’ is generally understood to refer to palliative care provided by non-specialist palliative care teams.^3–5^ The core competencies related to the palliative care approach have previously been elaborated and include, among others, early identification of persons requiring palliative care, screening for needs that affect a person’s quality of life (including physical, psychological, social, spiritual, and religious and cultural needs), initiating care plans to address these needs (including referring to specialist palliative care teams when needed), advance care planning and undertaking essential conversations such as goals of care discussions and family conferences, navigating ethical challenges, and collaborating with other professions and disciplines.^6^ Lack of these competencies leads to deficiencies in these various areas.

However, significant gaps exist in the provision of palliative and EOL care in LTC and several barriers impede the integration of palliative care in this setting.^7–10^ The lack of core palliative care skills among LTC staff remains a major impediment to providing a palliative approach in LTC homes.^11–13^ Access to timely, high-quality palliative care in LTC homes calls on all staff to have these core competencies.^14,15^

The COVID-19 pandemic highlighted palliative and EOL care gaps, especially as a disproportionate number of deaths occurred in LTC homes.^16–18^ Among others, lack of advance care planning, suboptimal serious illness conversations, poor symptom control, reduced physician visits, and lonely deaths resulting from social restrictions were reported.^17,19–24^ Healthcare professionals and other LTC staff experienced high levels of exhaustion, burnout, illness, and moral distress related to EOL care as they felt unable to adequately address residents’ needs.^18,25,26^ This has amplified previous calls to improve palliative care in LTC, including the training of staff.^27–30^ Standards and indicators related to palliative care training and preparedness of staff, legislation in this regards, and inclusion of palliative care in undergraduate and postgraduate curricula across the care professions are all strategies to increase readiness to provide a palliative care approach in LTC facilities.

Pallium Canada is a non-profit foundation established in 2000 to build primary-level palliative care capacity across different settings in Canada. Its approach, evolution, strategies, and challenges, and the instruction design of its suite of courses, called Learning Essential Approaches to Palliative Care (LEAP), have been previously reported.^31,32^ The short courses of 12–15 h aim to equip healthcare professionals across professions (including physicians, nurses, social workers, pharmacists, nursing aides, among others) across different care settings with the core competencies to allow them to provide a palliative care approach. There are different LEAP versions, including LEAP-LTC, which trains LTC staff on the palliative care approach. The courses largely use interprofessional, interactive, case-based learning and rely on a train-the-trainer model to spread.^32–36^ As part of an evaluation of the course to assess its impact and whether it is achieving its goals, the study aimed to explore the impact of the classroom version of the interprofessional LEAP-LTC course on physicians and nurses 2–4 years after they had completed the training. Thus said, the primary objective is to better understand the impact of taking a LEAP-LTC course on learners several years post-course. This study is the first investigation to assess learner impact for this specific course.

## Study design and methods

### Intervention

Study participants attended LEAP-LTC training as 2-day in-person classroom sessions. This version is 15 h long with a maximum of 25 participants in each course and targets different professions, including physicians, nurses, personal support workers, pharmacists, and social workers. It uses a modular design (Table 1). Participants in this study included only those who had participated in the classroom course. The impact of the LEAP-LTC Online course is being studied separately.

**Table 1.** LEAP-LTC course modules.

Module	Examples of topics
Being aware	Self-awareness, self-care, and peer support related to providing end-of-life care
Taking ownership	Defining palliative care, identifying residents with palliative care needs, initiating palliative care early, an overview of the palliative care approach, the role of palliative care specialist teams, and the respective roles of LTC staff and specialist palliative care teams (collaboration), the needs of residents.
Decision-making	Factors to consider when developing care plans, collaborative decision-making, a framework to guide ethical decision-making, and navigating ethical end-of-life challenges, illness trajectories.
*Pain*	Symptom assessment in general in residents with and without cognitive capacity, assessing and managing pain (in cancer and non-cancer situations).
*Delirium and dementia*	Screening and managing delirium and its different clinical forms, and distinguishing it from dementia.
*Essential conversations*	Advance care planning, goals of care discussions, initiating palliative care, addressing cultural needs of residents, family conferences, providing family support.
*Psychosocial and spiritual care*	Identifying and addressing psychological, social, and spiritual and religious needs of residents, dignity care, supportive counseling, non-pharmacological, and pharmacological support.
*Hydration, nutrition, and gastrointestinal symptoms*	Role of hydration and artificial nutrition in advanced frailty, dementia, and illnesses.
*Respiratory symptoms*	Identifying and managing breathlessness in cancer and non-cancer illnesses.
*Last days and hours*	Preparing for end-of-life, preparing families, medication reviews, managing airway secretions.
*Grief and bereavement care*	Identifying normal *versus* abnormal grieving and providing psychosocial support.
*Organizational readiness and quality improvement*	Opportunities to undertake palliative care-related quality improvement initiatives in a LTC home. Connecting to local palliative care resources.

LEAP, Learning Essential Approaches to Palliative Care; LTC, long-term care.

### Study design and methods

We sought an in-depth understanding of learner experiences of providing care. A qualitative descriptive study approach was used to guide the study design.^37^ This approach supports an understanding of people’s experiences, for example, providing care in LTC in the context of having formal palliative care education. Data were collected from a sample of care providers who had previously taken the LEAP-LTC course. The interview data were analyzed using a two-stage inductive analysis and an iterative approach of data collection (Stage 1). Framework method was then applied to group similar levels of impact at a micro, meso, and macro level (Stage 2).^38,39^ Rigor was supported through traditional qualitative techniques stated below. Ethical approval was received from Hamilton Integrated Research Ethics Board (HiREB # 14182).

Physicians, nurses, nurse practitioners, and registered practice nurses working in LTC homes in Ontario, Canada in 2021 who completed a LEAP-LTC course 2–4 years previously (2017–2019 inclusive) were invited to participate in a semi-structured interview. Email invitations were sent to potential participants through Pallium Canada’s alumni database, and by the Ontario Long-Term Clinicians Association to its members. Purposive sampling was utilized to ensure participants had taken the course during the specified time frames. Participants signed an emailed consent form; this was reviewed and confirmed before the interview. Recruitment continued until data saturation was reached and no new experiences relevant to the research question were identified.^40^

A palliative care physician trainee with interviewing experience collected data using an interview guide. Questions and probes explored LTC overall, impact of the LTC course on staff competencies, interactions, and teamwork. Interviews took place from May to June 2021, during the COVID-19 pandemic, and recorded on Zoom Video Communications (San Jose, California)™. Recordings were transcribed verbatim. Upon completion of data transcription, research staff read transcripts to ensure accuracy of transcription and replaced names of people and/or institutions to ensure anonymity. Proper nouns that could have identified study participants and/or their places of work were replaced with placeholders, such as ‘physician’ or ‘acute care hospital’ to ensure participants and their experiences could not be identified. Transcripts were uploaded to NVivo Rev 2020 (Lumivero, Denver, Colorado)™ to aid data management, coding, and analysis.

Data were coded inductively by three team members and described with labels to group similar feedback together. Inductive coding analysis was used in the first stage to identify categories addressed by participants. These categories were summarized early in the analysis process and agreed upon by team members by consensus discussion.^41,42^ When all data had been coded, members of the analysis team met and applied content analysis to determine how experiences were similar (or different) across learners.^41^ Using a Framework Method, a data matrix was generated from the content analysis coding and summarized as per micro, meso, and macro levels of impact.^38^ The three levels were individual (micro), facility-wide (macro), and a larger community (meso) aspects of care delivery.^39^ To ensure rigor was maintained during data collection and analysis, methodological congruence ensured that the techniques used in the study design fit together.^43,44^ Other techniques included bracketing, reflexivity, audit trails, and consensus meetings that drew upon analytic and clinical expertise from multidisciplinary team members.^44^ Both bracketing and reflexivity occurred during data collection and analysis. Interviewers completed field notes after each interview to reflect on their own impressions of data collection, and potential impact for data analysis. Debriefing between study team members during data collection and analysis further helped reflexivity to ensure researchers were describing learner experiences and not providing additional interpretation, to ensure no bias was introduced. Lastly, team meetings to review coding and data analysis helped to further debrief and review data, to ensure learner impact was assessed objectively and reflected the lived experiences of learners. Audit trails and making extensive interview and coding notes were reviewed during these meetings to ensure research staff were following planned data analysis and results were reflective of research goals and objectives.

## Results

Ten healthcare professionals participated: four registered practical nurses, three registered nurses, one nurse practitioner, and two physicians. Most participants were greater than 40 years of age, female, and working within a non-profit LTC home that were multi-chain.

**Figure 1.**
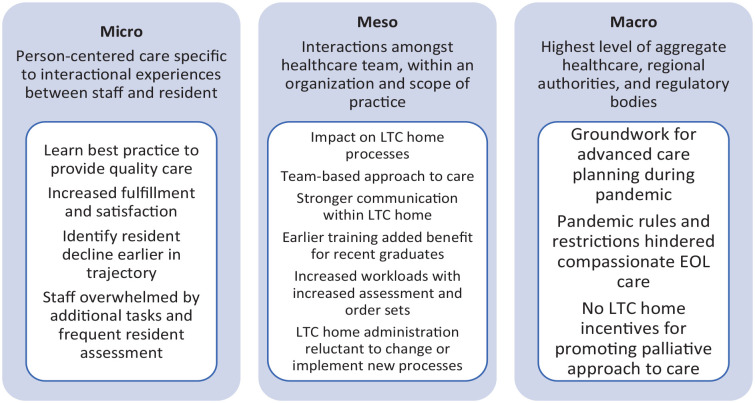
Levels of impact framework. Source: Adapted from Krawczyk (2019).

Learner experiences have been identified and organized according to their levels of impact at micro-, meso-, and macro-levels (Figure 1). A more-detailed description of impact at the three levels and supporting quotes can be referred to in Supplemental Material A.

### Micro-level impact

Participants described several ongoing benefits from the course. The training gave them increased knowledge and confidence to provide better palliative and EOL care. This included earlier identification of residents’ palliative care needs, more timely reviews of care plans and initiation of EOL care, improved advance care planning and EOL discussions, and more family engagement and conferences. The training provided confidence in providing EOL care, including communicating more effectively with residents, families, and colleagues. Additionally, participants suggested care approaches to other team members, including physicians. Participants described increased sense of work satisfaction and job fulfillment through better palliative and EOL care delivery.

So, I think [the course] increased confidence in my ability to care properly for a patient at end-of-life. . .Not burden. I think confidence has helped. Fulfillment, yes. I feel like our ability to provide proper and timely communication with family’s has really helped to avoid – I don’t want to say, poor deaths, let’s say deaths in something like an emergency room and that kind of thing which has helped us to feel good about what we’re doing. We often get compliments from families saying, ‘Thank you so much for your care.’ [P008]

Some participants reported frustration and even moral distress resulting from not being able to apply what they learned because of resistance from managers and other colleagues who had not undertaken palliative care training. Existing inadequate staffing levels were further exacerbated by the COVID-19 pandemic.

So, the palliative approach, unfortunately, and that end-of-life care you want to give, was very trying and hard for staff because of being so short staffed, so many very sick people as well that you were dealing with. So, and even the usual things you would do when somebody died and having an honor guard and our practices that we have in the home, you couldn’t even do that. So, it was a cold good-bye as well because you couldn’t honour that person and the time you spent with them because you were dealing with people who were so sick and dying and no staff, so it was hard. [P006]

### Meso-level impact

Training helped create a culture shift toward more acceptance and preparation to provide a palliative care approach within homes, especially when large groups of staff were trained. One manifestation was earlier and more widespread advance care planning and goals of care discussions in the home, the adoption of palliative care order sets and EOL checklists, and providing ‘palliative comfort kits’ to residents and families. Culture change was further facilitated by support from management, particularly if they had learned about palliative care approach themselves, and the presence of ‘palliative care champions’. Increased interprofessional collaboration and communication across staff was described, such as an environment of shared responsibilities with resident assessment and symptom monitoring. All levels of staff (from house-keeping and personal support workers to nurses and physicians) engaged in assessing and monitoring patients and identifying decline in function or change in status and communicating these to the rest of the team. There was more consistent messaging to family from staff, and increased team discussions regarding patient care. The pressure to initiate a conference or communicate with family was no longer all on the nurse.

The course was an opportunity to network and us all feel part of a team that we’re all – have a role even though in our facility we try to foster that we’re a team. . . . I could reassure the families that we’re a team and that the team has been trained and the team can be there with their families especially when we had all the restrictions of COVID . . . [P002]

A ripple-effect was noted: staff who had undergone training would sometimes teach colleagues in the home who had not received training on the palliative care approach. Some participants described fewer residents being transferred out of the homes to acute care hospitals at EOL because of the training. However, some participants explained best practices in a palliative care approach sometimes resulted in increased workloads, including increased assessments, and monitoring of residents, especially at the EOL. It also required more communication with residents and families. However, the root cause of this challenge was described as pre-existing workforce shortages further exacerbated by the COVID-19 pandemic.

So, when nurses have let’s say, three or four – let’s say, out of the 30 residents, four of those residents are palliative care. . . . its time consuming for this particular nurse to do all these things to also call the other family members and give them updates . . . and take care of the unit, take care of staffing needs, it becomes overwhelming. [P004]

Training appeared to help some participants and their teams respond to the COVID-19 pandemic. They felt equipped to undertake advance care planning and goals of care discussions, communicate with families, address symptoms, and prioritize care. However, some noted there was little that prepared them for the unprecedent impact of the pandemic. They described feeling overwhelmed and distressed as teams, unable to provide what they knew to be high-quality palliative and EOL care that came from the training.

### Macro-level impact

Macro-levels of impact extended across homes and external partners. LEAP-LTC attendees engaged with other LTC homes, hospices, and communities of practice, to help coordinate responses and learn from each other, especially during the pandemic. Participants exercised advocacy roles for palliative care approach in their units and facilities, as well as the LTC system at large.

The value of palliative care training for all staff and the need to incorporate palliative care education in early career training to better integrate palliative care across the whole sector was highlighted. Staff commented that new graduates had little or no experience in EOL care; the pandemic exacerbated this lack of experience through increased workloads, reduced staff, and social restrictions.

I had a student who had never seen someone at end-of-life before and didn’t know how to care for them. So, I had to slowly explain the process and stuff but in a pandemic it’s – like the way end-of-life works, you don’t really have time to stop and try to explain to someone while you’re in an outbreak and you’re dealing with end-of-life. So, I feel like maybe more education now that we know that we’ve had a pandemic, like what we should do in this case. [P001]

System-level barriers impacted the ability to implement new skills and processes. These include absence of appropriate quality indicators for palliative care (including palliative care training of staff). For example, typical quality indicators include wound care, number of falls, and improved behavioral symptoms. These performance indicators identified by funding and accreditation bodies sometimes challenge the integration of palliative care approach in LTC as there are no ‘incentives’ to adopt important processes and outcome measures.

Quality indicators do not include palliative care, end-of-life care. So, . . .. when it comes down to funding and making your home look good, palliative care is not part of that. . . And it’s really sad because I’ve worked in long-term care for 20 years and you start a program, you do an education like our LEAP program, you do this education, you’re all pumped and you’re doing really good and ‘Oh, oh, our wounds are bad, the percentage is high, . . . corporate says we have to do this. . .’ so, out goes palliative care. [P007]

## Discussion

In this study, participants across varying professions described ongoing benefits to taking palliative care training and acquiring core palliative care skills to provide a palliative care approach. These benefits were identified 2–4 years after completing a relatively short, 2-day course. Training was found to have impacted them as individuals – largely through increased skills and confidence to provide a palliative care approach and enhanced sense of work satisfaction and fulfillment – as well as the teams and homes in which they worked. This included increased integration of palliative care in the homes through improved advance care planning and goals of care discussions, improved symptom assessment and management, implementation of palliative and EOL protocols and checklists, and increased support of family through strategies such as comfort kits. Examples of impact on patients and the homes were described, including improved communication and collaboration among team members, improved engagement with and support of residents and families, and reduced transfer of residents to hospitals at the EOL.

The results are encouraging in that they align with the key goals, competencies, and learning objectives described for the LEAP-LTC course. Moreover, the impact for the participants of this course, were long term (2–4 years) after completing the training. There is evidence, using the commitment-to-change approach, of learners implementing what they learned following participation in a LEAP Core (primary care teams) up to 4 months after participating in the course.^45^ This study provides evidence of longer-term impact, at least for some learners. It is also reassuring that the impact was described across the different professions. LEAP-LTC training also resulted in increased work satisfaction and fulfillment, and interprofessional collaboration, likely attributable to increased confidence and self-efficacy, gained from new knowledge and skills to provide palliative and EOL care as learnings were put into practice.^33^ Increased work satisfaction has been expressed by paramedics and primary care providers who participated in other LEAP course versions.^33,34,45^ The impact of LEAP-LTC training is largely consistent with the impact reported in other LEAP course versions (including those targeting primary care providers, paramedics, and cancer care professionals) and by other training programs.^34,35,45–48^

The LEAP courses helped get the whole team on the ‘same page’, and also improved interprofessional collaboration’.^35,36^ For some, training also resulted in frustrations ascribed to a lack of palliative care training of LTC staff and managers, staff shortages, and quality indicators that did not align with good palliative and EOL care practices. A recent update for LTC in Canada is absent specific performance indicators related to a palliative care approach. These frustrations have been also reported in other studies^1,9–11,49^ and the need for implementing improvements have been noted as well. The sense of increased work satisfaction and fulfillment and interprofessional collaboration are noteworthy, particularly at a time when the LTC sector is facing considerable workforce attrition and LTC staff are experiencing unprecedented burnout. The need to increase care provider experiences has been highlighted as one of four quality improvement goals in healthcare, as articulated by the Quadruple Aim.^50,51^ Equipping LTC staff with core palliative care skills increased their confidence and work–place satisfaction and may have a protective role.^52^

Implications for practice and research includes an urgent call for more palliative care education across the health professions, in pre-certification curricula and continuing education for healthcare staff.^8–10,13,25,53,54^ The new Fixing Long-Term Care Act in Ontario mandates the provision of quality palliative care services for all LTC residents, which will require ensuring appropriate training for all LTC staff.^55^ Furthermore, additional research is warranted to better understand learner experiences for LEAP-LTC, and delivery of the course itself. Such investigations will also explore the impact of classroom *versus* online delivery of such educational sources, a delivery change required during COVID-19, and part of a current LEAP evaluation.

This study has three key limitations. Those who volunteered for the study valued the palliative care approach and may have been inclined to overstate course impact. Second, participants were asked to reflect on past LEAP-LTC education during an on-going pandemic. Unpacking LEAP education, skills, and experiences from those demanded by the pandemic was a challenging request at times. Lastly, the study did not include personal support workers who provide a large proportion of care in LTC homes. Pallium Canada has a separate course called LEAP Personal Support Worker (PSW) for these workers, including nursing aides; this will be studied separately. Notwithstanding these limitations, the results provide useful insights into the impact of palliative care approach training and align with previous findings in this and other care settings.

## Conclusion

Palliative care education and building primary palliative care capacity in LTC homes are critical elements of integration of palliative care in LTC. Short-intensive training of LTC staff can impart benefits to residents, families, the staff themselves, as well as the LTC homes. These benefits can continue long after course-end. For successful adoption and implementation of palliative care approach, it is essential to have institutional readiness, and external and internal incentives and support for implementing new processes. Large-scale investments by government, LTC homes, and the healthcare system is urgently needed to support rapid upskilling on palliative care approach competencies in this sector. Education programs like LEAP-LTC exist and are ready for large-scale deployment.

## Supplemental Material

sj-docx-1-pcr-10.1177_26323524241235180 – Supplemental material for ‘Confidence and fulfillment’: a qualitative descriptive study exploring the impact of palliative care training for long-term care physicians and nurses Supplemental material, sj-docx-1-pcr-10.1177_26323524241235180 for ‘Confidence and fulfillment’: a qualitative descriptive study exploring the impact of palliative care training for long-term care physicians and nurses by Ashlinder Gill, Lynn Meadows, Jessica Ashbourne, Sharon Kaasalainen, Sandy Shamon and José Pereira in Palliative Care and Social Practice

sj-docx-2-pcr-10.1177_26323524241235180 – Supplemental material for ‘Confidence and fulfillment’: a qualitative descriptive study exploring the impact of palliative care training for long-term care physicians and nurses Supplemental material, sj-docx-2-pcr-10.1177_26323524241235180 for ‘Confidence and fulfillment’: a qualitative descriptive study exploring the impact of palliative care training for long-term care physicians and nurses by Ashlinder Gill, Lynn Meadows, Jessica Ashbourne, Sharon Kaasalainen, Sandy Shamon and José Pereira in Palliative Care and Social Practice

## Declarations

**Ethics approval and consent to participate:** The study was approved by the Hamilton Integrated Research Ethics Board (HiREB #13177). All study participants provided informed consent.

**Consent for publication:** Informed consent included consent for the utilization of anonymized quotes for publication.

**Availability of data and materials:** Deidentified and anonymized transript data may be available upon request, in the form of theme summary reports.
