# Supplementary material for: ‘Confidence and fulfillment’: a qualitative descriptive study exploring the impact of palliative care training for long-term care physicians and nurses
Source: Palliat Care Soc Pract. 2024 Mar 5;18:26323524241235180. doi: 10.1177/26323524241235180 (PMC10916492; doi:10.1177/26323524241235180)
Supplement: sj-docx-2-pcr-10.1177_26323524241235180 – Supplemental material for ‘Confidence and fulfillment’: a qualitative descriptive study exploring the impact of palliative care training for long-term care physicians and nurses [file sj-docx-2-pcr-10.1177_26323524241235180.docx]

| Level of Impact | Theme | Illustrative quotes |
| --- | --- | --- |
| MICRO: person-centered care specific to interactional experiences between healthcare provider and patient/family member. | *Best practice, better quality of care:* learning best practice for specific medications, and symptom management, such preferred doses/medications. Updated recommendations provided more clarity around misconceptions. For more experienced learners, this was a refresher to help ground current understanding.  *Increased fulfillment and job satisfaction:* better quality of care delivered. Death can be a positive experience, with a shift from curative to comfort intent of care.  *Ability to identify patient decline* *earlier in their trajectory:* initiate family meeting/conference earlier for increased planning and preparedness, implement order sets when appropriate. LTC specific context was helpful  *Better able to connect and engage with families:* increased comfort to discuss patient decline. Learn the language/tone/body language with increased knowledge about communication with families. Able to provide options, and additional resources.  *Pocketbook* was helpful resource, especially case studies and summaries.  *Staff limited capacity:* overwhelmed with tasks, and difficult to keep up with palliative orders, constant reassessing of patient, and maintaining consistent messaging with care team and family (more heightened due to pandemic). | *“I would say, “Not burden. I think confidence has helped. Fulfillment, yes.” I feel like our ability to provide proper and timely communication with family’s has really helped to avoid—I don’t want to say, poor deaths, let’s say deaths in something like an emergency room and that kind of thing which has helped us to feel good about what we’re doing. We often get compliments from families saying, “Thank you so much for your care.” So, I think with the course background increased confidence in my ability to care properly for* a patient at end-of-life…” [P008] |
| MESO: interactions specific to the healthcare team, and within the healthcare organization and scope of practice. | *Impact on the entire LTC home*: use of palliative care kits (e.g. coloring activity, lip balm, personal care items). Shift in culture, to facilitate change (e.g. reluctance to prescribe opioids, palliative considered very end-of-life). Change easier to implement with medical director/staff being on the “same page” with palliative approach to care (PAC), and having a local champion, someone who has your back with implementing new processes. LEAP LTC presents various tools/approaches to help facilitate change with providing greater palliative approach to care.    *Team-based palliative approach to care*: All levels of staff (e.g. house keeping, PSW, RN, MD) engaged in assessing patient’s function, documenting any decline in function, and then discussion with broader care team/family about care planning earlier in trajectory. Collaboration amongst colleagues encouraged, discussing medications/doses, from increased collective knowledge from LEAP LTC at the micro level. Providers felt more confident and comfortable to engage in such discussions. Increased connected with peers and colleagues, as external resources (e.g. partner LTC homes, hospice, community of practice), and resident families. Shared responsibility amongst all staff to notice change in function early in resident’s trajectory, where pressure isn’t all on the RN to initiate pall care conference or communicate with family.  *Communication within LTC home strengthened*: Increased collaboration and interdisciplinary approach to managing resident’s care/needs. Consistent messaging to family from all staff, with team-based approach to care.  *Increased workloads and demands:* Palliative care approach requires increased time from staff, to maintain EOL orders, consistently reassessing patient function, maintaining communication with families. Need additional staff/resources to relieve staff and send for training/education.  *LTC home culture and acceptance of palliative approach to care*: reluctance from providers to implement palliative orders or engage in processes to identify palliative care needs, reluctance to prescribe opioids. Enforcing change can be slow and/or difficult. | *Well, what medication would you think would work better?” And before, I’d be like, “You’re the doctor.” (Laughs) “Why are you asking me,” type of thing. But now I understand why they’re asking. They’re like, “Do you think this—recently, I spoke to a doctor and they’re like, “We have an as needed medication that we can give them like every so often, and we have this routine medication that’s only every like this many… I think we should make the routine medication more frequent because we’ll still have the ‘as needed’ medication but this also forces us to go and look at the patient, because obviously in a COVID pandemic, it’s obviously harder to be everywhere all at once because there’s so many reasons. And depending on your floor might be heavy in certain areas like, you want to be able to check on them and I can go give my pain medication or I can go assess them and then I can come back—and it’s more for comfort and we came up with this plan with certain medications. We were like, “Okay, we’ll schedule this medication this often, more frequently than it was,” and we added a range to cover which was nice. So, the family definitely appreciated it and I know it was much easier for me to talk to the doctor about this medication whereas before I’d be like, “I don’t know, why are you asking me?” (Laughs)* [P001]  *Yeah, and that’s why I say, “The course was an opportunity to network and us all feel part of a team that we’re all—have a role even though in our facility we try to foster that we’re a team. But knowing and meeting some of the people and knowing that they’ve been a part of the training also. I could reassure the families that we’re a team and that the team has been trained and the team can be there with their families especially when we had all the restrictions of COVID and that often the team will be a family replacement and that they have the training to offer comfort and symptom control to the last day.* [P002]  *so they can hear us, and they hear the language, they hear the family accept end-of-life and then they’re a lot more comfortable to answer those calls when they say, “Yeah, my mom’s changing.” “Oh, yes you know you had that talk with the team, and we talked about how this would happen and blah, blah, blah.” So, I think it’s really helped them feel more comfortable and less burdened with being the only one who’s talked to the family*. [P005]  *“It’s a course that is telling you this is how this person is declining,” it is when you have to start seeking extra help… but there’ve been times where maybe this person has to be sent out to… But when we took the course, there was something that happened. Most of the nurses felt like providing palliative care is such a task. I’m not saying that it’s—the truth is providing palliative care to a human being from the nurse’s perspective is such a huge task. More especially here in the long-term care where you have 30 residents, you have to give medication to 30 residents. You have CHF use… and you have this resident that is constantly, constantly waiting for you to go in check, check him head to toe and give the medications or asks for more medications or this medication maybe we need to add something else. Yeah. It’s like it’s an eye opener. You open your eyes to a lot of things. Through the course, I was able to say, “Okay, is this the time that we need to call the doctor?” So, when nurses have let’s say, three or four—let’s say, out of the 30* *residents, four of those residents are palliative care. Of course, you have to give your morphine, you have to give your hydro morphine, you have to give your Haldol, you have to give all these meds. If its time consuming for this particular nurse to do all these things to also call the other family members and give them updates and give updates to these ones and take care of the unit, take care of staffing needs, it becomes overwhelming.* [P004]  *I think it’s like—Train the Trainer though as well when we had a PSW there and they could be giving that hands-on training and feedback to the individual staff. We revised our ‘Palliative End-of-Life’ checklist as well. So, that’s when somebody is palliative and you start the checklist to say, “Okay, is there enough chairs in the room for the family? Do you have music? And what kind of music do the family—would like to listen to? Have you offered them tea and coffee?” It’s all on this checklist so that you can address everything that you’d need to address.* [P006]  *Oh, I think so. And that’s—right there is the problem. I mean, you have people coming back from the palliative care course and with these ideas and common sense idea I feel there, and then you take that back to your institution and like right from the top down, you could have them say, “Well, no that’s not—this isn’t going to happen.” We have like for instance say, “The palliative room,” we have a palliative room on the Alzheimer unit, somebody came and changed that into a just a patient’s room. And I said, “Well, you can’t do that.” (Laughs) “You can’t do that, that’s—that room is for Alzheimer patients.” “Well, we need that bed.” I said, “No! This is designed for that,” and you have to start that fight all over again… LEAP program, CAPCE program, they’re all amazing. I think that everyone everywhere should take those courses. But unless you have people that are coming into the programs with some knowledge, any knowledge of palliative care, it’s a fight. It’s always going to be a fight.* [P008] |
| MACRO: highest level of aggregate healthcare for organizing responses to structural and social determinates of health at the population level. Government priority setting, regional authorities, and regulatory bodies. | *Earlier training added benefit:* Increased benefit to learners if offered earlier in training, a part of education/schooling for nursing, getting exposed to dying/death and how to interact with families. Newer staff are often inexperienced.  *Groundwork for EOL care discussions during pandemic:* foundations for care planning taught by the course, given every resident was asked about their EOL care preferences. However, modules or special seminars specific to outbreak management, emergency planning or infection control would have been an added benefit.  *Pandemic rules and restrictions*: increased staff turnover and often no background in EOL care provision, understaffing and unable to maintain symptom management and EOL order sets for all residents, and keep up with usual activities. Unable to have in-person meetings and training/education meetings.  *No “incentives” for Palliative Care*: performance indicators and measures are specific to ED transfers, wound care etc. but no specific funding/resources to allocate for palliative care provision. Having a *palliative approach* not reflected for administrative/ministry initiatives. | *I’ve said and I’ve said this all along, “If they don’t have this in curriculums for their school, you’re never going to get the palliative quality that you should get, unless you work in a palliative unit and even then, there’s issues but, if you don’t have students taking these courses, you’re never going to have what you’re looking for… And that’s why I’ve come to the realization that—well even when I had a chance to talk to some students and I said, “You have to go back to your schools and tell them, this has to be in your program. There is no way it shouldn’t be.” Any of the nursing programs like I said, “PSW,” otherwise you’re just going to be battling it all the time.* [P008]  *Well, they were able to identify like how to talk to family members, but for when it comes down to the Pandemic, personally I wasn’t prepared. I don’t think that’s something that course did mention at that point because as far as I can recall—because nobody saw this coming. So, maybe now more advocation on that part should be included because we have newer staff, we have because of the pandemic people are so rushed, they need to get a job, but then they don’t have—some people have never—I had a student who had never seen someone at end-of-life before and didn’t know how to care for them. So, I had to slowly explain the process and stuff but in a pandemic it’s—like the way end-of-life works, you don’t really have time to stop and try to explain to someone while you’re in an outbreak and you’re dealing with end-of-life. So, I feel like maybe more education now that we know that we’ve had a pandemic, like what we should do in this case. [P001]*  *So, the palliative approach, unfortunately and that end-of-life care you want to give was very trying and hard for staff because of being so short staffed, so many very sick people as well that you were dealing with. So. And even the usual things you would do when somebody died and having an honor guard and our practices that we have in the home, you couldn’t even do that. So, it was a cold good-bye as well because you couldn’t honor that person and the time you spent with them because you were dealing with people who were so sick and dying and no staff, so it was hard.* [P006]  *Quality indicators do not include palliative care, end-of-life care. So, if your home has to focus on a certain percentage, you’ve got to get this under control, and falls under control and all that stuff, when it comes down to funding and making your home look good, palliative care is not part of that… And it’s really sad because I’ve worked in long-term care for 20 years and you start a program, you do an education like our LEAP program, you do this education, you’re all pumped and you’re doing really good and “Oh, oh, our wounds are bad, the percentage is high, this is high, corporate says we have to do this…” so, out goes palliative care*. [P007] |
